# Role of Serum Fibrinogen Levels in Patients with Rotator Cuff Tears

**DOI:** 10.1155/2014/685820

**Published:** 2014-04-10

**Authors:** Umile Giuseppe Longo, Stefano Petrillo, Alessandra Berton, Filippo Spiezia, Mattia Loppini, Nicola Maffulli, Vincenzo Denaro

**Affiliations:** ^1^Department of Orthopaedic and Trauma Surgery, Campus Bio-Medico University, Via Alvaro del Portillo 200, Trigoria, 00128 Rome, Italy; ^2^Centro Integrato di Ricerca (CIR), Campus Bio-Medico University, Via Alvaro del Portillo 21, Trigoria, 00128 Rome, Italy; ^3^Centre for Sports and Exercise Medicine, Mile End Hospital, Mann Ward, 275 Bancroft Road, London E1 4DG, UK; ^4^Department of Musculoskeletal Medicine, University of Salerno, 84048 Salerno, Italy

## Abstract

Although rotator cuff (RC) tendinopathy is a frequent pathology of the shoulder, the real understanding of its aetiopathogenesis is still unclear. Several studies showed that RC tendinopathy is more frequent in patients with hyperglycemia, diabetes, obesity, or metabolic syndrome. This paper aims to evaluate the serum concentration of fibrinogen in patients with RC tears. Metabolic disorders have been related to high concentration of serum fibrinogen and the activity of fibrinogen has been proven to be crucial in the development of microvascular damage. Thus, it may produce progression of RC degeneration by reducing the vascular supply of tendons. We report the results of a cross-sectional frequency-matched case-control study comparing the serum concentration of fibrinogen of patients with RC tears with that of a control group of patients without history of RC tears who underwent arthroscopic meniscectomy. We choose to enrol in the control group patients with pathology of the lower limb with a likely mechanic, not metabolic, cause, different from tendon pathology. We found no statistically significant differences in serum concentration of fibrinogen when comparing patients with RC tears and patients who underwent arthroscopic meniscectomy (*P* = 0.5). Further studies are necessary to clarify the role of fibrinogen in RC disease.

## 1. Introduction

Rotator cuff (RC) tendinopathy is a frequent disorder of the shoulder, producing great healthcare costs in the industrialized countries and representing the most costly problem in Workers' Compensation Systems after low-back pain [1–4]. The incidence of RC tears ranges from 5% [5] to 39% [6], being the third cause of musculoskeletal disease (16%), after the spine (23%) and the knee (19%) [7].

The clear understanding of the aetiology and aetiopathogenesis of RC tendinopathy remains a challenge [8–11]. Combinations of intrinsic factors (age, gender), extrinsic factors (such as load, sport, and work), and metabolic factors have been described in the development of RC tears [12, 13].

The role of hyperglycemia as a risk factor for RC tear has been investigated [14]. Preliminary reports focused on the analysis of the nonenzymatic glycosylation process, which changes collagen cross-links causing tendon degeneration [15–17]. The presence of statistically significant higher level of fasting plasma glucose in nondiabetic patients undergoing arthroscopic RC repair has been already demonstrated [14]. Other authors showed an association between both type 1 and type 2 diabetes mellitus and chronic RC tendinopathy in men but not in women [18]. These findings confirm the fact that metabolic syndrome may play a role in RC tendinopathy and it may be relevant to predict which patients may have an increased risk of developing RC tendinopathy.

Some authors focused their attention on the correlation between serum levels of lipids and RC tears [19, 20]. The interest in this relationship arises from the potential role of high serum lipid concentration in complete rupture of the Achilles tendon [21, 22]. Fatty degeneration, or tendolipomatosis, was found in the histopathological examination of specimens harvested during surgery for tendinopathy in the lower limb [23]. However, similar results were not obtained from tendon samples of the RC [24] and the long head of the biceps [25]. In a previous study [26], no statistically significant difference in serum triglyceride and total cholesterol concentrations between patients undergoing arthroscopic RC repair and patients of a similar age undergoing arthroscopic meniscectomy has been reported. On the other hand, Abboud and Kim [19] observed higher levels of total cholesterol, triglycerides, low-density lipoprotein cholesterol, and lower levels of high-density lipoprotein cholesterol in patients with RC tears compared to patients with shoulder pain but without RC tears. Nevertheless, this data was not confirmed by histological/pathological evidence of cholesterol deposition. Consequently, no definitive conclusion on the role of serum cholesterol and triglyceride concentration in the pathogenesis of RC tears can be formulated.

Obesity can be considered another important risk factor for the development of RC tendinopathy. Overweight patients have elevated cholesterol, atherosclerosis, diabetes, hypertension, metabolic syndrome, and decreased physical activity. Since vascular supply is essential for the metabolic processes of the tendons, all these conditions associated with obesity or with increased body mass index (BMI) may represent a cause of decreased vascularity, interplaying in the onset and progression of RC tears. In a recent cross-sectional study [18], abdominal obesity was associated with chronic RC tendinopathy. Furthermore, both obesity and metabolic syndrome are associated with increased concentration of proinflammatory cytokines including IL-1, IL-6, and TNF*α* [27–32], as well as reactive oxygen species (ROS). Proinflammatory cytokines have been proved to be upregulated inrat and human models of RC tendinopathy [33]. In this respect, raised circulating IL-1, IL-6, and TNF*α* may aggravate shoulder complaints, for example, by maintaining inflammation. Moreover, they play a crucial role in the apoptosis process, particularly in that induced by oxidative stress, leading to tendon degeneration [34].

The aim of this cross-sectional frequency-matched case-control study was to compare the serum levels of fibrinogen in patients with RC tears and those in patients without history of RC tears who underwent arthroscopic meniscectomy and who were used as control group.

## 2. Materials and Methods

The study included 164 subjects (72 male and 92 female) who underwent arthroscopic RC repair or meniscectomy at our institution.

Group 1 (study group) included 82 patients (36 men and 46 women; mean age: 57.7 ± 10.2 years, range 29 to 76) who underwent arthroscopic repair of RC tears. The dominant arm was affected in 68 patients. The rotator cuff tears were classified as small (≤3 cm) in 32 patients, medium (3 ≥ 5 cm) in 36 patients, and large (>5 cm) in 14 patients. The tear involved the supraspinatus tendon in 37 patients; both supraspinatus and infraspinatus tendons in 31 patients; and both supraspinatus and subscapularis tendons in 14 patients (Table 1).

Group 2 (control group) included 82 patients (36 men and 46 women; mean age: 55.9 ± 9 years, range 30 to 73) who underwent arthroscopic meniscectomy for a meniscal tear with no history of RC symptoms. These patients were frequency-matched by age (within 3 years) and gender with patients of Group 1.

### 2.1. Inclusion Criteria

Patients in Group 1 were included in the study if they had RC tear diagnosed on clinical and imaging grounds and confirmed at the time of surgery. Conservative management, including nonsteroidal anti-inflammatory drugs, physiotherapy, and rest, failed in all patients, and they continued to experience unacceptable pain and weakness in the affected shoulder. None of the patients had undergone prior surgery on the affected shoulder. All patients fulfilled the following criteria: (1) positive rotator cuff lag signs on preoperative examination (at least one among Jobe test, Napoleon test, lift-off test, and Patte test), (2) no episodes of shoulder instability, (3) no radiographic sign of fracture of the glenoid or the tuberosities, (4) magnetic resonance imaging (MRI) evidence of cuff tear, (5) RC tear of 1 or more tendons at arthroscopic examination, and (6) no lesion of the glenoid labrum or of the capsule at arthroscopic examination.

Patients in Group 2 were included in the study if they had a meniscal tear diagnosed on clinical and imaging grounds and confirmed at the time of surgery.

### 2.2. Exclusion Criteria for All Participants

Patients were excluded in case of primary osteoarthritis of the operated or contralateral shoulder, previous operations on the shoulder or knee, and inflammatory joint disease.

Patients in Group 2 were also excluded from the study if they have had history of shoulder pain or rotator cuff pathology diagnosed by imaging or on clinical grounds.

### 2.3. Measurement of Serum Fibrinogen Levels

All blood samples were collected in an identical manner between 07.00 and 07.30 after an overnight fast started at 12.00 midnight. Samples were collected into a plastic or siliconized glass tube, 9 parts of freshly drawn venous blood and 1 part of trisodium citrate 3.8%. The plasma was separated after centrifugation of the mixture for 10 minutes at 1500 ×g. The determination of fibrinogen with thrombin clotting time was performed using the method originally described by Clauss (in the presence of an excess of thrombin, fibrinogen is transformed into fibrin and clot formation time is inversely proportional to the concentration of fibrinogen in the sample plasma).

### 2.4. Statistics

Data were entered in a commercially available database. Descriptive statistics were calculated, and analytical statistics were performed with the unpaired sample* t*-test using Statistical Programs for the Social Sciences (SPSS). *P* values lower than 0.05 were considered statistically significant.

## 3. Results

The serum levels of fibrinogen were measurable in all patients. When comparing the two groups, no statistically significant differences in serum concentration of fibrinogen were present (*P* = 0.5) (Table 1). Besides, we were not able to determine any statistically significant differences in serum concentration of fibrinogen in patients with small, medium, or large RC tears. Equally, there were no statistically significant differences in serum concentration of fibrinogen in patients with a supraspinatus tendon tear or supraspinatus and infraspinatus tendons' tear or supraspinatus and subscapularis tendons' tear.

Therefore, for the purposes of this study, all tears were grouped together.

### 3.1. Group 1

In Group 1 (study group), the mean fibrinogen serum concentration was 335.9 ± 171 mg/dL (range 70–512; median 328.5) (Table 2). Fibrinogen concentration was higher than 400 mg/dL in 15 (18%) patients (5 male; 10 female) (Table 1).

Of these patients, 6 (40%) had a small or medium tear, while 3 (20%) patients had a large tear. In 7 (47%) patients, the RC tear involved the supraspinatus tendon or both supraspinatus and infraspinatus tendons, while in 1 (6%) patient the RC tear involved both supraspinatus and subscapularis tendons (Table 3).

### 3.2. Group 2

In Group 2 (control group), the mean fibrinogen serum concentration was 329.6 ± 205 mg/dL (range 72–607; median 322.5) (Table 2). Fibrinogen concentration was higher than 400 mg/dL in 10 (12%) patients (1 male; 9 female) (Table 1).

## 4. Discussion

Following the evidences on the relationship between metabolic disorders and RC tendinopathy and taking into account that high serum levels of fibrinogen have been described in different metabolic disorders in which has been demonstrated also an increased incidence of RC tears [34], such as obesity [35], metabolic syndrome [36], hyperglycemia, and diabetes [37], we hypothesized that the serum concentration of fibrinogen could be a predictor factor for the onset of RC tendinopathy.

In the present study, which is a cross-sectional frequency-matched case-control study, the serum levels of fibrinogen obtained from patients who underwent arthroscopic RC repair were compared with the serum levels of fibrinogen obtained from a control group of patients of a similar age who underwent arthroscopic meniscectomy. To our knowledge, this is the first study on this topic.

Patients with RC tear showed no statistically significant difference in serum fibrinogen concentrations compared to subjects of the same age and sex undergoing arthroscopic meniscectomy who had no history of RC injury. However, the serum concentration of fibrinogen was higher in patients with RC tears compared to patients of the control group. Moreover, no statistical significant relationship has been found between extension of the RC tear, as well as RC tendon involved, and serum concentration of fibrinogen.

The activity of fibrinogen has been proven to be crucial in the development of microvascular damage. High concentration of serum fibrinogen determines an increased viscosity of the plasma producing erythrocyte and platelet aggregation, impairing vascular contractility and endothelial integrity with a consequent damage of the microcirculation. High levels of fibrinogen may ultimately produce progression of RC degeneration reducing the vascular supply of tendons.

However, despite several studies [34] showing that RC tears are more frequent in patients with obesity, diabetes, metabolic syndrome, and hyperglycemia and all these pathologies are related to high concentration of serum fibrinogen, the serum concentration of fibrinogen does not correlate with an increased incidence of RC tears.

Although several advances have been made in the surgical management of RC tears, the aetiology of RC tendinopathy is still unclear, and its understanding is fundamental because RC tears represent an important healthcare problem producing elevated costs in the industrialized countries. Combinations of intrinsic (age, gender) and extrinsic factors (such as load, sport, and work), as well as biological factors, were described in the development of injury of the RC.

Intrinsic factors include injuries to the RC via tensile overload, aging, or microvascular supply through traumatic, reactive, or degenerative insults to the tendons [24, 25, 38]. Extrinsic factors include injuries to the RC through compression of the tendons by bony impingement or direct pressure from the surrounding soft tissue [39] or microtrauma [24]. Anyhow, genetics and biological factors play a role in RC pathology. Siblings of patients diagnosed with full thickness RC tears had more than twice the relative risk for developing a lesion and nearly five times the risk of experiencing symptoms than spousal controls [40, 41]. Furthermore, the correlation between tendinopathy and serum levels of some substances was largely demonstrated. The role of hyperglycemia as a risk factor for RC tear has been investigated [14]. We have already showed that statistically significant higher fasting plasma glucose levels within the normoglycemic range have been found in nondiabetic patients undergoing arthroscopic RC repair, compared with patients of a similar age undergoing arthroscopic meniscectomy [14]. Patients with RC tear are likely to have hypercholesterolemia when compared with a control group [42]. We could not find similar results in our population of patients with RC tears and our data suggest no role of the serum cholesterol and triglyceride concentration in RC tears.

Strengths of the present study include the systematic collection of blood samples, the use of preoperative imaging and arthroscopy to diagnose RC and meniscal tears, and the relatively large sample size of our study group. Nevertheless, we acknowledge the cross-sectional nature of the present investigation, which cannot completely resolve issues concerning temporality or rule out other factors that may influence RC tendinopathy. Another limitation of our study was that the control group did not include healthy people. Among the various diseases of the lower limb, we choose to enroll in the control group patients with pathology of the lower limb with a likely mechanic, not metabolic, cause, different from tendon pathology.

On the basis of our study, we think that fibrinogen serum levels do not correlate with RC tears, even though further research is necessary to reach definitive conclusion.

## 5. Conclusions

Patients with metabolic disorders such as metabolic syndrome, diabetes, obesity, hyperglycemia, and dyslipidemia have an increased incidence of RC tears or tendinopathy when compared with healthy population. However, the molecular processes underlying the onset of RC tendinopathy are still unclear.

The role of serum lipids in RC tendinopathy remains unclear since studies on the topic are discordant. Actually, no definitive conclusion on the role of serum cholesterol and triglyceride concentration in the pathogenesis of RC tears can be made, and further studies are necessary to clarify the issue.

We hypothesized that the serum concentration of fibrinogen may be the link between metabolic disorders and RC tears. In the present study, the serum concentration of fibrinogen was higher in patients with RC tears when compared with patients in the control group. However, this difference was not statistically significant. Moreover, no statistical significant relationship has been found between extension of the RC tear, as well as RC tendon involved, and serum concentration of fibrinogen. On the basis of our study, we doubt that fibrinogen serum levels have a causative role in the pathogenesis of RC tears, even though we advocate more research to reach a definitive conclusion.

## Figures and Tables

**Table 1:** High fibrinogen serum levels.

	Rotator cuff damage	Controls	*P*
Patients with elevated FNG	15 (5 male; 10 female)	10 (1 male; 9 female)	0.5

**Table 2:** Comparison of fibrinogen serum levels.

Serum fibrinogen values	Group 1 (study group)				Group 2 (control group)			
	Male		Female		Male		Female	
	mg per decilitre	Millimoles per litre	mg per decilitre	Millimoles per litre	mg per decilitre	Millimoles per litre	mg per decilitre	Millimoles per litre
Mean	317.6	0.093	350.3	0.103	311.2	0.091	343.9	0.016
Median	320.5	0.094	353.5	0.104	307	0.09	329.5	0.017
SD	70	0.02	67	0.019	57	0.016	79.6	0.002
Range	171–512	0.05–0.1	238–498	0.019–0.14	211–435	0.062–0.127	205–607	0.009–0.021

**Table 3:** Extension of RC tear: RC tendon involved and fibrinogen serum levels.

	High FNG	Normal FNG
Extent of RC damage <3 cm	6 (40%)	26 (39%)
Extent of RC damage 3–5 cm	6 (40%)	30 (45%)
Extent of RC damage >5 cm	3 (20%)	11 (16%)
Supraspinatus	7 (47%)	24 (35%)
Supraspinatus + infraspinatus	7 (47%)	30 (45%)
Supraspinatus + subscapularis	1 (6%)	13 (20%)
